# In Situ Electrospinning Iodine-Based Fibrous Meshes for Antibacterial Wound Dressing

**DOI:** 10.1186/s11671-018-2733-9

**Published:** 2018-10-03

**Authors:** Guo-Sai Liu, Xu Yan, Fang-Fang Yan, Fu-Xing Chen, Long-Yun Hao, Shao-Juan Chen, Tao Lou, Xin Ning, Yun-Ze Long

**Affiliations:** 1 Industrial Research Institute of Nonwovens and Technical Textiles, College of Textiles and Clothing, Qingdao University, Qingdao, 266071 China; 2 Collaborative Innovation Center for Eco-Textiles of Shandong Province, Qingdao University, Qingdao, 266071 China; 3 College of Chemistry and Chemical Engineering, Qingdao University, 308 Ningxia Road, Qingdao, 266071 China; 4 Collaborative Innovation Center for Nanomaterials and Optoelectronic Devices, College of Physics, Qingdao University, Qingdao, 266071 China

**Keywords:** In situ electrospinning, PVPI fibrous meshes, Wound dressing

## Abstract

For effective application of electrospinning and electrospun fibrous meshes in wound dressing, we have in situ electrospun poly(vinyl pyrrolidone)/iodine (PVP/I), PVP/poly(vinyl pyrrolidone)-iodine (PVPI) complex, and poly(vinyl butyral) (PVB)/PVPI solutions into fibrous membranes by a handheld electrospinning apparatus. The morphologies of the electrospun fibers were examined by SEM, and the hydrophobicity, gas permeability, and antibacterial properties of the as-spun meshes were also investigated. The flexibility and feasibility of in situ electrospinning PVP/I, PVP/PVPI, and PVB/PVPI membranes, as well as the excellent gas permeabilities and antibacterial properties of the as-spun meshes, promised their potential applications in wound healing.

**Electronic supplementary material:**

The online version of this article (10.1186/s11671-018-2733-9) contains supplementary material, which is available to authorized users.

## Background

Thanks to the advantages of easy large-scale production, huge surface-area-to-volume ratio, high porosity, and tunable inner structures [1–4], electrospun fibrous meshes have attracted a lot of interests in various fields such as filtration [5, 6], medical care [7–12], and energy [13, 14]. Electrospun fibrous membranes are suitable for wound dressing due to their nanoscale structures which mimic the collagen fibrils of the native extracellular matrix and human organs [9, 11], and then, the as-spun meshes can not only physically protect the wound from contaminants and infections, but also provide an ideal environment for skin regeneration through maintaining an adequate exchange of gases, as well as promoting hemostasis phase and avoiding scar induction [9, 11, 12].

Among the thousands of suitable electrospun materials, poly(vinyl pyrrolidone) (PVP) and poly(vinyl butyral) (PVB) are two important polymers for their excellent biocompatibility, nontoxicity, good solubility in alcohol, and so on [15–18]. Consequently, the as-spun PVP and PVB fibrous materials have been popularly applied for wound dressing [18–20]. Moreover, PVP in combination with iodine forms a complex called PVP-iodine (PVPI) and has been a highly efficient and widely used disinfectant for its small stimulation, low toxicity, light pollution, broad-spectrum bactericidal effect, and nonresistance of the microorganisms for even longtime using [21–24]. Nevertheless, PVPI is not recommended for long-term use or for complex wounds [25]. Electrospun PVP-I-based fibers may be a helpful solution and have been reported by several groups [26–33]. Ignatova et al. had prepared PVPI or poly(ethylene oxide) (PEO)/PVP-I fibers by directly electrospinning PVPI or PEO/PVP-I solutions or by crosslinking PVP and PEO/PVP mats and treating them with iodine vapor or iodine solution [26]. Wang had fabricated PVPI nanofibers by electrospinning PVP, iodine, and absolute ethanol solutions, and the characterization of as-spun fibers from infrared spectra, Raman spectra, and X-ray diffraction ensures the formation of PVPI complex [27]. Uslu et al. have reported series of PVPI-based electrospun fibers such as poly(vinyl alcohol) (PVA)/PVPI [28], PVA/PVPI/poly(ethylene glycol) (PEG) fibers containing (hydroxypropyl)methyl cellulose (HPMC) and aloe vera [29], PVA/PVPI nanofibers with additional chitosan and poloxamer 188 [30], and PVA/poly(acrylic acid) (PAA)/PVPI fibers [31]. All these PVPI fibers were known to show potential applications in wound dressing, however, mostly focused on the morphologies and thermal stability of the as-spun fibers/meshes. Hong et al. have reported PLLA/PVPI/TiO_2_ multicomponent ultrathin fibrous nonwovens by electrospinning and iodine vapor treatment [32]. It was found that the existence of PVPI endowed the nonwoven with water absorbability, antimicrobial activity, adhesive ability, and transformable characteristic from hydrophilicity to non-hydrophilicity. Sebe et al. have prepared PVP/poly(vinylpyrrolidone-vinylacetate)/iodine nanofibers with different polymer ratios by a high-speed rotary spinning technique. Except for the detailed morphological analysis, the supramolecular structure and antimicrobial activity of the obtained mats were also investigated, which suggested the potential applications in wound dressing [33]. However, for practical applications, these PVPI electrospun fibers can only be fabricated based on predesigned models and then implanted onto the patient wound, which may lead to the second injuries to the wound. In situ electrospinning might solve this problem.

In this paper, we have in situ electrospun iodine-based PVP and PVB solutions into fibrous meshes by a hand-held portable electrospinning apparatus. The morphology, hydrophobicity, gas permeability, and antibacterial property of the as-spun meshes were examined. Moreover, the effects of iodine concentrations on these properties were also investigated. Furthermore, the flexibility and feasibility of in situ electrospun iodine-based fibrous mats were presented, and then, the application for wound dressing can be expected.

## Methods/Experimental

### Materials

Polyvinylpyrrolidone (PVP, 250 kDa, Sinopharm Chemical Reagent Co., Ltd., China) was dissolved in ethanol (Sinopharm Chemical Reagent Co., Ltd., China) at 13 wt%. Poly(vinyl butyral) (PVB) (100 kDa, Sinopharm Chemical Reagent Co., Ltd., China) was dissolved in ethyl alcohol at 10 wt%. Iodine (Analytical reagent, Sinopharm Chemical Reagent Co., Ltd., China) was added into PVP/ethanol solutions at concentration of 1 wt%, 2 wt%, and 5 wt%, respectively. Poly(vinylpyrrolidone)-iodine complex (PVPI, Sinopharm Chemical Reagent Co., Ltd., China) was dissolved in the PVP/ethanol and PVB/ethanol solutions at 1 wt%, 2 wt%, and 5 wt%, respectively. The complex solutions were agitated at room temperature under constant stirring for at least 24 h before electrospinning. Modified simulated body fluid (SBF) was purchased from Sinopharm Chemical Reagent Co., Ltd., China.

### Electrospinning Process

**Fig. 1:**
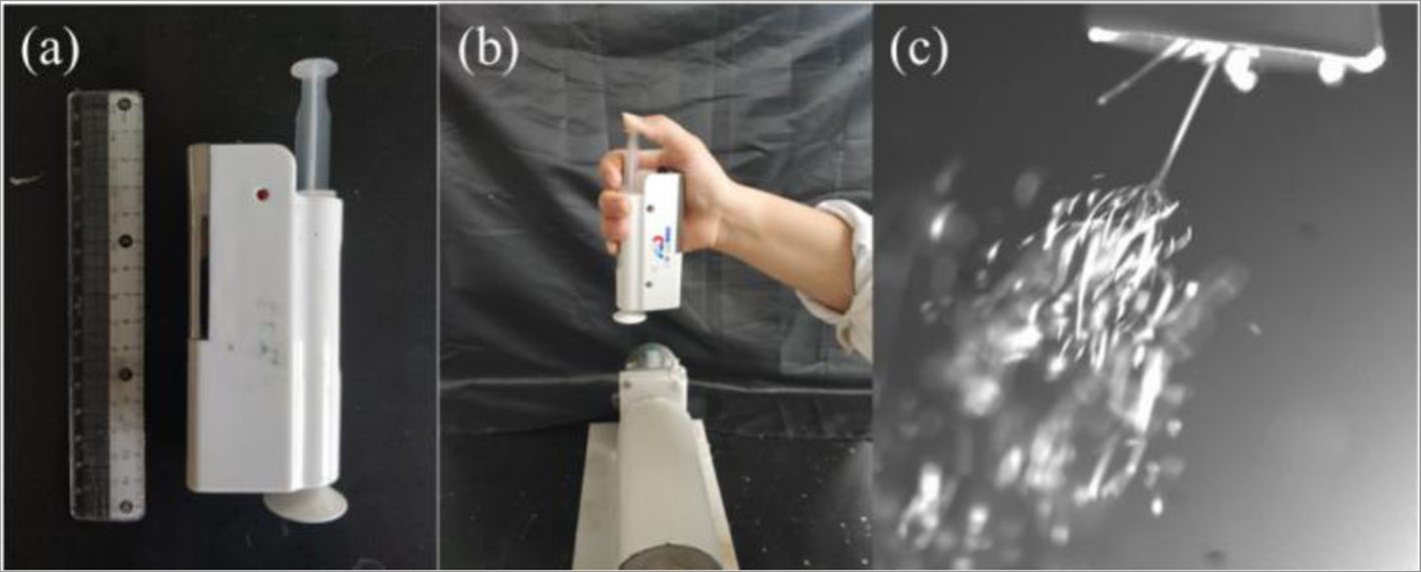
The handheld electrospinning apparatus (**a**) and the in situ electrospinning process (**b**). The electrospinning jets can be seen from the spinneret (**c**)

The prepared solutions were placed into a 5-mL syringe equipped with a nozzle with a diameter of 0.1 mm, and then loaded into the hand-held portable electrospinning apparatus (HHE-1, Qingdao Junada Technology Co., Ltd), as shown in Fig. 1a. The high voltage of this device is about 10 kV fixed [34, 35]. During the in situ electrospinning process, one can firstly operate the device and then press the syringe by a finger. The as-spun fibers can be fabricated and then deposited onto the collector, as suggested in Fig. 1b. The electrospinning jets by this device can be caught by a high-speed camera, which is shown in Fig. 1c. For the further examinations of the in situ electrospun fibrous meshes, we also in situ electrospun these fibers onto an aluminum foil collector with distance of 8 cm. The collected meshes were uncovered from the aluminum foil for further characterization.

### Characterization

The morphology and energy dispersive system (EDS) of the as-spun fibers were examined by a scanning electron microscope (SEM, Phenom ProX, Phenom Scientific Instruments Co., Ltd., China) at 10 kV, and all samples were coated with gold for 30 s before analysis. The Fourier transform infrared spectroscopy (FTIR) spectrums were measured by a Thermo Scientific Nicolet iN10 spectrometer. The simulated body fluid (SBF) contact angle was examined by a Contact Angle Analyzer (JY-PHb, China) with a 2-μL SBF droplet. Based on ASTM D 737 standard, the air permeability under a pressure drop of 200 Pa was tested by an air permeability tester (Textest FX3300). Pore sizes of the as-spun fibrous meshes were examined by PSM 165 (Germany, Topas GmbH, PSM 165) at pressure of 200 Pa. The antibacterial properties of the as-spun meshes were investigated against *Escherichia coli* (*E. coli*, ATCC 10536) and *Staphylococcus aureus* (*S. aureus*, ATCC 25923) bacteria. Bacterial cells of *E. coli* (ATCC 10536) and *S. aureus* (ATCC 25923) were grown for 24 h on a shaker at 37 °C and 100 rpm.

## Results and Discussion

### Morphologies of Electrospun Fibers

**Fig. 2:**
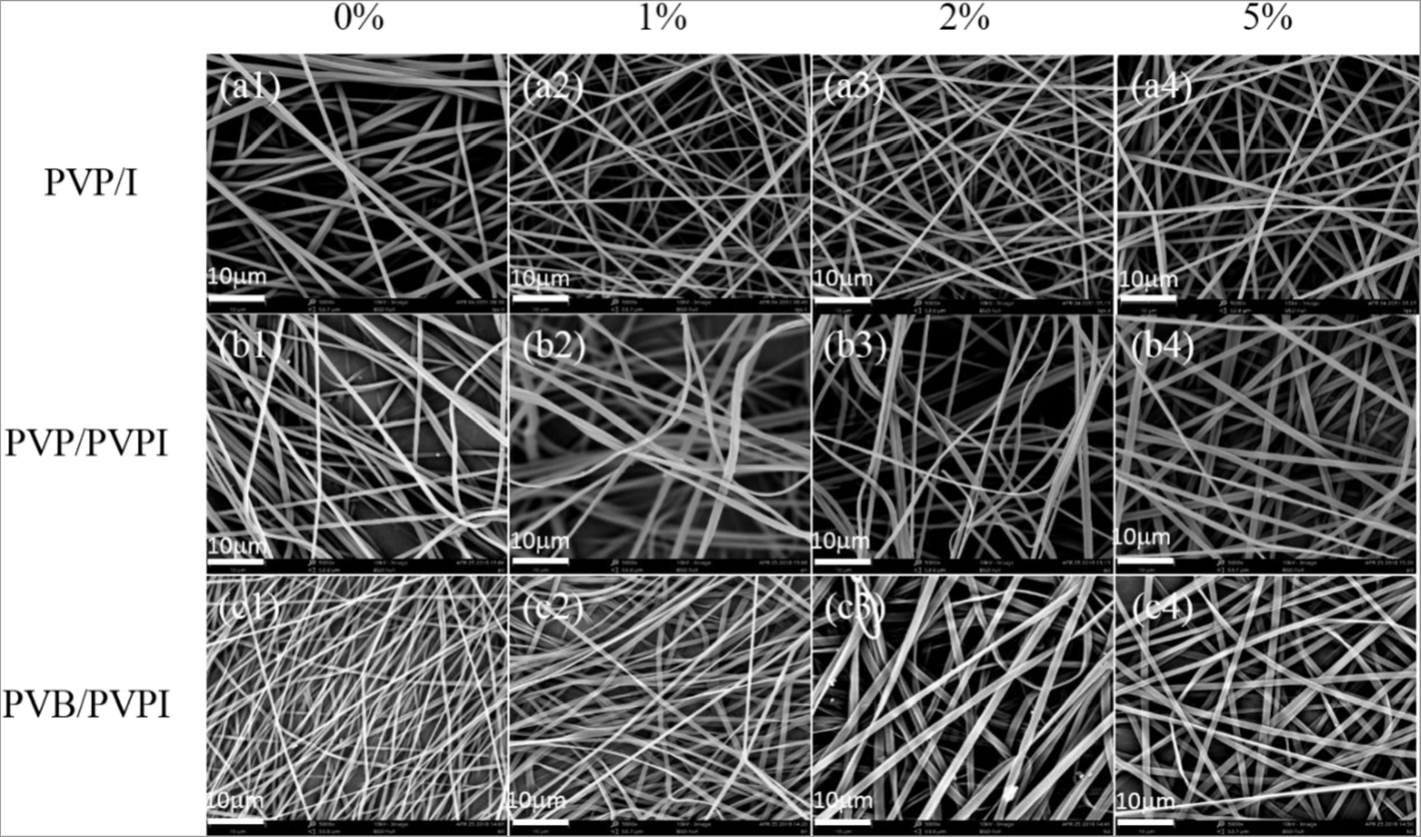
SEM images of the as-spun PVP/I (a1–a4), PVP/PVPI (b1–b4), PVB/PVPI (c1–c4) fibers with I or PVPI concentrations of 0%, 1%, 2%, and 5%, respectively

By the HHE-1 apparatus as shown in Fig. 1, the prepared PVP/I, PVP/PVPI, and PVB/PVPI solutions can be electrospun into fibers conveniently. The morphologies of the as-fibers could be found from the SEM images shown in Fig. 2. From the SEM images, one can obviously find that the electrospun fibers displayed smooth surfaces, while the diameters of the as-spun fibers showed different distributions because of the different materials and concentrations. Combining SEM images and the data in Table 1 comprehensively, it is shown that for PVP/I fibers, as the concentration of iodine increasing, the average diameter of the as-spun fibers decreased obviously, which may due to the higher conductivity of the solutions as iodine is added [36]. While for PVP/PVPI and PVB/PVPI, the average diameters of the as-spun fibers were both increased with higher concentration of PVPI, which may result from the increasing of viscosities of the mixed solutions [37].

**Table 1:** The average diameter of PVP/I, PVP/PVPI, and PVB/PVPI fibers with different doping concentrations of iodine and PVPI, with unit of nanometer

Materials	0%	1%	2%	5%
PVP-I	857 ± 14	852 ± 259	724 ± 132	511 ± 134
PVP-PVPI	857 ± 14	948 ± 89	1092 ± 216	1445 ± 351
PVB-PVPI	523 ± 81	849 ± 194	1231 ± 332	1485 ± 242

### EDS and FTIR

**Fig. 3:**
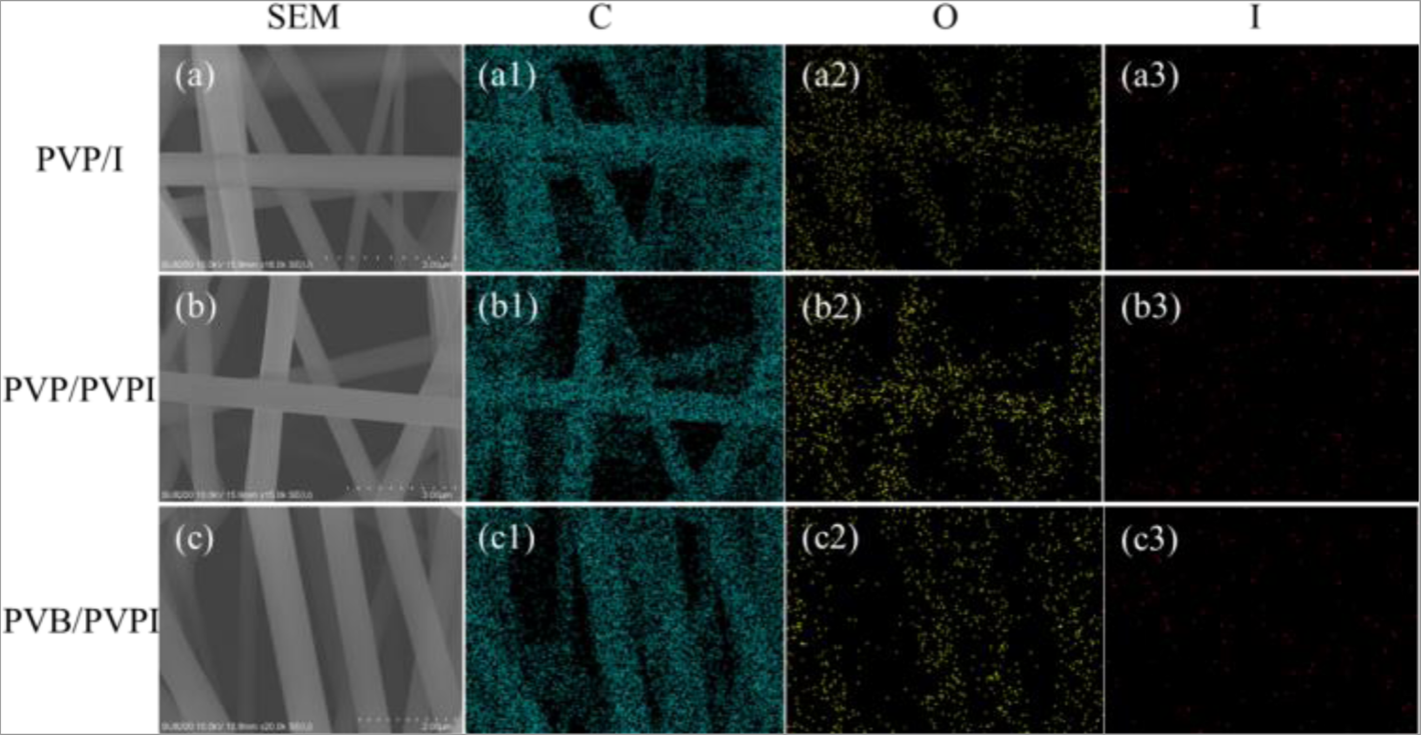
Different elements of EDS images of the as-spun PVP/I (a–a3), PVP/PVPI (b–b3), PVB/PVPI (c–c3) fibers with 5% I/PVPI doping

**Fig. 4:**
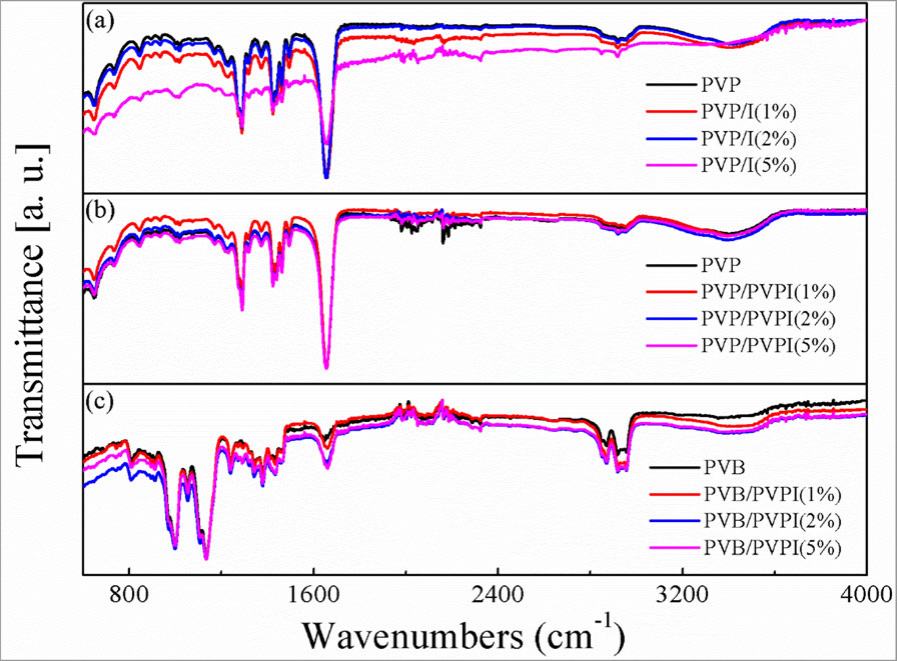
FTIR spectra of the as-spun fibers PVP/I (**a**), PVP/PVPI (**b**), PVB/PVPI (**c**)

To achieve the antibacterial properties and then benefit the wound healing application, iodine played the crucial role in the electrospun fibers. To verify the existence of iodine, EDS was examined in the model of full spectrum analysis. As displayed in Fig. 3, we chose the as-spun fibers with higher concentrations of I/PVPI, 5%, for example, and the images showed that in each kind of electrospun fibers, except for the mainly carbon (Fig. 3 (a1), (b1), and (c1)) and oxygen (Fig. 3 (a2), (b2), and (c2)) elements in the polymers, extra iodine element was also observed (Fig. 3 (a3), (b3), and (c3)). Moreover, the iodine added into the PVP solutions directly showed a high concentration of iodine other than PVPI added. Although the iodine could be found in the EDS images, one can obviously find from Fig. 3 that the content of iodine is small compared with other elements. The same conclusion can be obtained from the FTIR spectra in Fig. 4.

Fig. 4a–c showed the FTIR spectra of the as-spun fibers with different concentration of different additions. As can be seen from Fig. 4, the additions of iodine or PVPI do not change the chemical structures of the polymers obviously, which may due to the small quantity of the additions. The unchanged polymers also ensured the stability of the polymers for wound healing, without any other uncertainties.

### Wettability

**Fig. 5:**
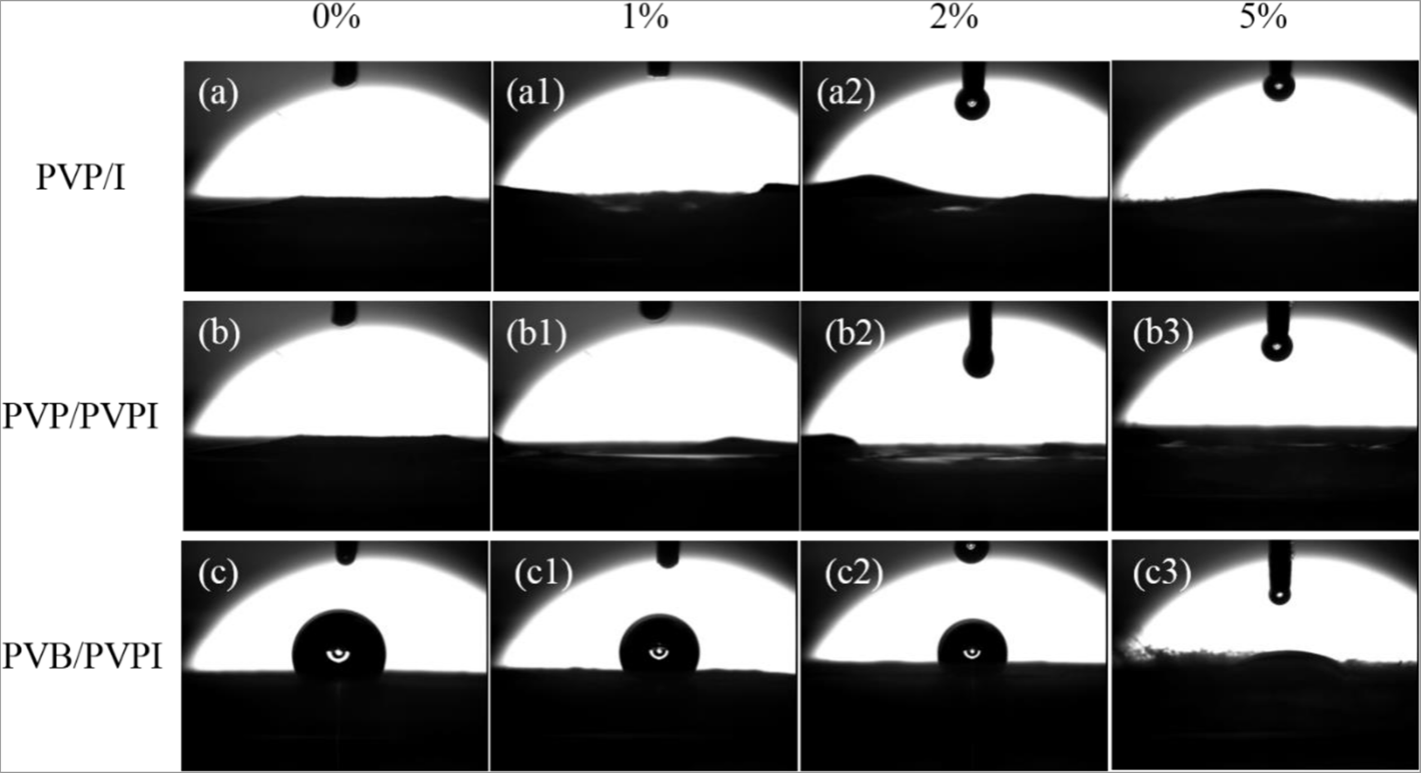
SBF contact examination of the as-spun fibers PVP/I (a–a3), PVP/PVPI (b–b3), PVB/PVPI (c–c3) with different iodine/PVPI concentrations

Furthermore, it was believed that an ideal wound dressing should include some advantages such as maintenance of wound hydration and absorption of excess wound exudate, which may require the wettability of the designed wound dressing [5, 7–9]. Consequently, we also examined the hydrophilicity of the as-spun fibrous meshes by measuring their SBF contact angles. As suggested in Fig. 5, the three kinds of electrospun fibrous membranes all exhibited good hydrophilicity with the increasing concentration of iodine and PVPI. For PVP-based meshes, due to the hydrophilicity of the polymer, the electrospun fibrous meshes also established small SBF contact angles, and the angle increased to 19.5° for PVP/I, as shown in Fig. 5 (a–a3) and (b–b3). The increased SBF contact angles may result from the increasing surface roughness of these meshes. However, the case in PVB-based meshes was different. In our previous study, it had been pointed out that electrospun PVB fibrous meshes showed hydrophobicity due to its unequal structures [38]. In the absence of PVPI, the PVB electrospun meshes showed the similar contact angle case as can be seen in Fig. 5(c). As PVPI is doped in PVB, the SBF contact angle decreased and rapidly to zero with PVPI higher than 2%, which indicated that PVPI increased the hydrophilicity of the as-spun fibrous meshes. The good hydrophilicity of these fibrous meshes ensured the ability of absorption of excess wound exudate and then would be beneficial for wound dressing applications.

### Air Permeability

An ideal wound dressing also requires good air permeability to provide a positive environment for wound healing [9, 11–13]. Here, we also investigated the air permeability of these kinds of iodine-doped fibrous meshes, as shown in Table 2. As can be found in Table 2, with the increasing doping of iodine in PVP, the air permeability was also increased from 59.92 to 324.3 mm s^−1^, which may result from the decreased diameter and increased porosity, while the air permeability of fibrous meshes with PVPI doped in PVP and PVB does not show obviously trends. Nevertheless, the 5% doping ones show better gas permeability than the pure polymers. For comparison, we also tested the air permeability of two traditional wound dressings (TWD) bought from the market. It is clear that the designed electrospun fibrous wound dressings establish better air permeability than the ones on the market.

**Table 2:** Air permeability of the electrospun fibrous mats and two kinds of TWD (traditional wound dressing), with unit of mm s^−1^

Materials	0%	1%	2%	5%
PVP/I	59.92 ± 8.51	68.3 ± 12.87	95.68 ± 4.83	324.3 ± 31.74
PVP/PVPI	59.92 ± 8.51	143 ± 16.83	89.93 ± 7.12	73.19 ± 2.64
PVB/PVPI	44.99 ± 5.54	21.66 ± 2.60	72.08 ± 7.75	74.16 ± 7.41
TWD 1	8.45 ± 1.56	–	–	
TWD 2	17.82 ± 2.12	–	–	

For further examination of the air permeability, we tested the pore size and pore distribution of the as-spun meshes. As shown in Table 3, the average pore sizes of the as-spun meshes were listed. Generally, the bigger the average pore size, the better air permeability, compared with the data in Table 2. Moreover, the pore sizes of the as-spun fibrous meshes were mainly uniform, with the largest portion at the mean sizes, which can be found in Additional file 1: Figure S1. The pore sizes of these electrospun meshes were in the region of 1.936–9.152 μm, matching the human tissue cells sizes, which would be beneficial for wound healing. However, due to the instrument precision, the pore sizes of the TWD were too small to be tested, which may result in the poor air permeability of them.

**Table 3:** Average pore sizes of the electrospun fibrous mats, with unit of micrometer

Materials	0%	1%	2%	5%
PVP/I	2.357 ± 0.395	2.831 ± 0.634	4.353 ± 1.211	9.152 ± 2.274
PVP/PVPI	2.357 ± 0.395	5.996 ± 2.306	5.185 ± 0.904	4.274 ± 1.174
PVB/PVPI	3.732 ± 0.964	1.936 ± 0.518	3.792 ± 1.366	4.786 ± 1.192

### Antibacterial Activity

**Fig. 6:**
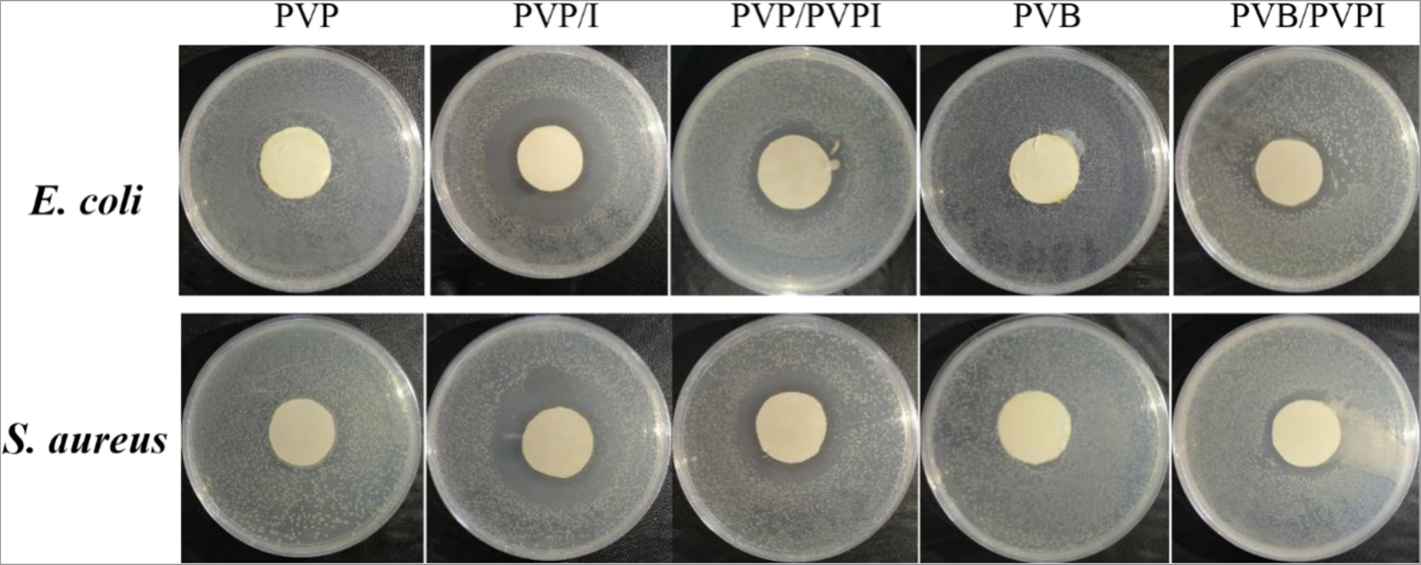
The antibacterial activity of the as-spun membranes against *E. coli* and *S. aureus*

Another requirement for ideal wound dressing is asepsis and even antibiosis to prevent and treat wound infections [11–13]. In this work, the iodine and PVPI doping are rightly to achieve that. The antibacterial activities of the as-spun fibrous meshes were assessed against typical pathogenic bacteria, such as *E. coli* and *S. aureus*, as displayed in Fig. 6. From Fig. 6, one can find that no bacteriostatic circle was formed for pure PVP or PVB. Once iodine or PVPI was doped in the polymer, the as-spun fibrous membranes showed obvious inhibition zones for the two bacterial strains after 24 h intervals. Moreover, the iodine-doped PVP showed the best antibacterial properties against both *E. coli* and *S. aureus*, the PVPI-doped PVP taking the second place and PVB/PVPI last. The good antibacterial properties ensured that the iodine-based electrospun fibrous meshes could be used for wound healing against bacterial infections of the wound. Moreover, it can be expected that the higher the concentration of the additional antibacterials, the better the antibacterial properties of the meshes. Consequently, one can easily get better antibacterial properties by adding more iodine or PVPI in their solutions.

### In Situ Applications

**Fig. 7:**
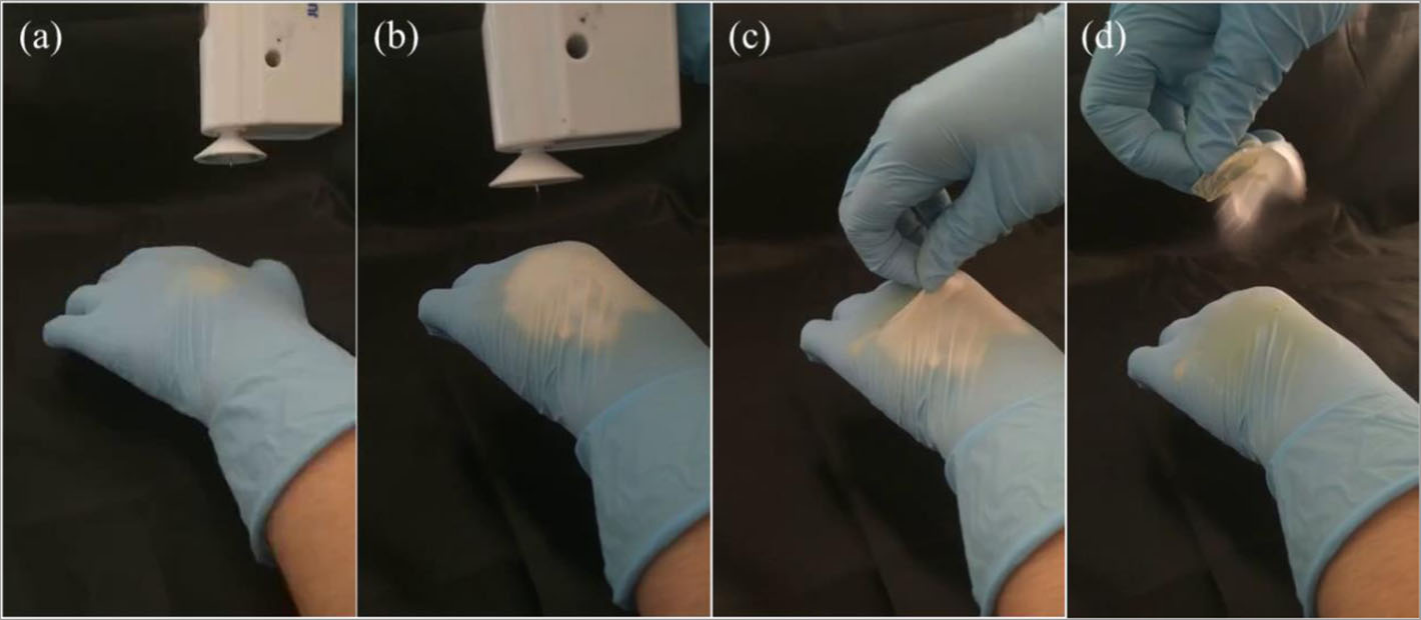
In situ applications of the handheld apparatus and iodine-based electrospun fibrous mats. By the HHE-1, one can easily in situ electrospun iodine-based PVP/I mesh onto the “injured hand” (**a**–**b**), the electrospun mats can be easily removed from the “wound bed” (**c**–**d**)

It is believed that in situ wound dressing will benefit their efficiency due to additional superiority such as conformability without wrinkling or fluting in the wound bed, ease of application, and improved patient compliance and comfort [39]. Consequently, in situ electrospinning is considered as a useful concept to produce appropriate substitutes for tissue repairing and wound healing directly on the patient’s lesion independently of wound size and depth [18, 34, 35, 40, 41]. As shown in Fig. 7a, b, the iodine-based fibrous meshes can be in situ electrospun onto the “injured hand” by the HHE-1 device and form a thin film on the surface of the skin like a second layer of skin due to electrostatic attraction forces. The electrospun PVP-I fibrous membrane shows good flexibility and compactness and can be easily removed if needed [see Fig. 7c, d). The more vivid details of in situ electrospinning of PVP-I wound dressing can be found in Additional file 1: Video S1 and S2 and Figure S2.

## Conclusions

In summary, we have in situ electrospun PVP/I, PVP/PVPI, and PVB/PVPI into fibrous membranes by a hand-held electrospinning apparatus. These electrospun meshes show uniform diameters and better hydrobilicity with doping of iodine or PVPI. Moreover, the good air permeability of blend PVP/I, PVP/PVPI, and PVB/PVPI electrospun meshes ensures their application in wound dressing. The increased concentrations of iodine and its complex favor the antibacterial properties of these meshes and then improve the effects as wound dressing. Furthermore, the in situ electrospinning also benefits the electrospinning process and the as-spun fibrous meshes for wound healing.

## Additional File

Additional file 1: **Figure. S1.** Pore size distribution of the as-spun PVP/I (a-a3), PVP/PVPI (b-b3) and PVB/PVPI(c-c3) fibrous mats with concentration of I/PVPI 0%, 1%, 2% and 5%, respectively. **Figure S2.** Electrospun PVP-I meshes onto human injured finger, can stem the bleeding quickly, and then heal the wound well. **Figure S3.** In situ electrospun PVP/I meshes onto human hand and finger, the as-spun meshes showed good conformability on the finger. (ZIP 24007 kb)

## Abbreviations

*E. coli*: *Escherichia coli*
EDS: Energy dispersive system
FTIR: Fourier transform infrared spectroscopy
HPMC: (Hydroxypropyl)methyl cellulose
PAA: Poly(acrylic acid)
PEG: Poly(ethylene glycol)
PEO: Poly(ethylene oxide)
PVA: Poly(vinyl alcohol)
PVB: Poly(vinyl butyral)
PVP: Poly(vinyl pyrrolidone)
PVP/I: Poly(vinyl pyrrolidone)/iodine
PVPI: Poly(vinyl pyrrolidone)-iodine
*S. aureus*: *Staphylococcus aureus*
SEM: Scanning electron microscope
WCA: Water contact angle (WCA)
