# Supplementary material for: In Situ Electrospinning Iodine-Based Fibrous Meshes for Antibacterial Wound Dressing
Source: Nanoscale Res Lett. 2018 Oct 3;13:309. doi: 10.1186/s11671-018-2733-9 (PMC6170247; doi:10.1186/s11671-018-2733-9)
Supplement: Supplementary file 1 — Figure. S1. Pore size distribution of the as-spun PVP/I (a-a3), PVP/PVPI (b-b3) and PVB/PVPI(c-c3) fibrous mats with concentration of I/PVPI 0%, 1%, 2% and 5%, respectively. Figure S2. Electrospun PVP-I meshes onto human injured finger, can stem the bleeding quickly, and then heal the wound well. Figure S3. In situ electrospun PVP/I meshes onto human hand and finger, the as-spun meshes showed good conformability on the finger. (ZIP 24007 kb) [file 11671_2018_2733_MOESM1_ESM.zip › Supporting information-revised.docx]

**Supporting information**

***In situ* electrospinning iodine-based fibrous meshes for antibacterial wound dressing**

Guo-Sai Liu^1^, Xu Yan*^1,2^, Fang-Fang Yan^1^, Fu-Xing Chen^1^, Long-Yun Hao^1,2^, Shao-Juan Chen*^1,2^, Tao Lou^3^, Xin-Ning^1,2^, Yun-Ze Long^1,2,4^

^1^ Industrial Research Institute of Nonwovens & Technical Textiles, College of Textiles & Clothing, Qingdao University, Qingdao 266071, China

^2^ Collaborative Innovation Center for Eco-Textiles of Shandong Province, Qingdao University, Qingdao 266071, China

^3^ College of Chemistry & Chemical Engineering, Qingdao University, 308 Ningxia Road, Qingdao, 266071, PR China

^4^ Collaborative Innovation Center for Nanomaterials & Optoelectronic Devices, College of Physics, Qingdao University, Qingdao 266071, China


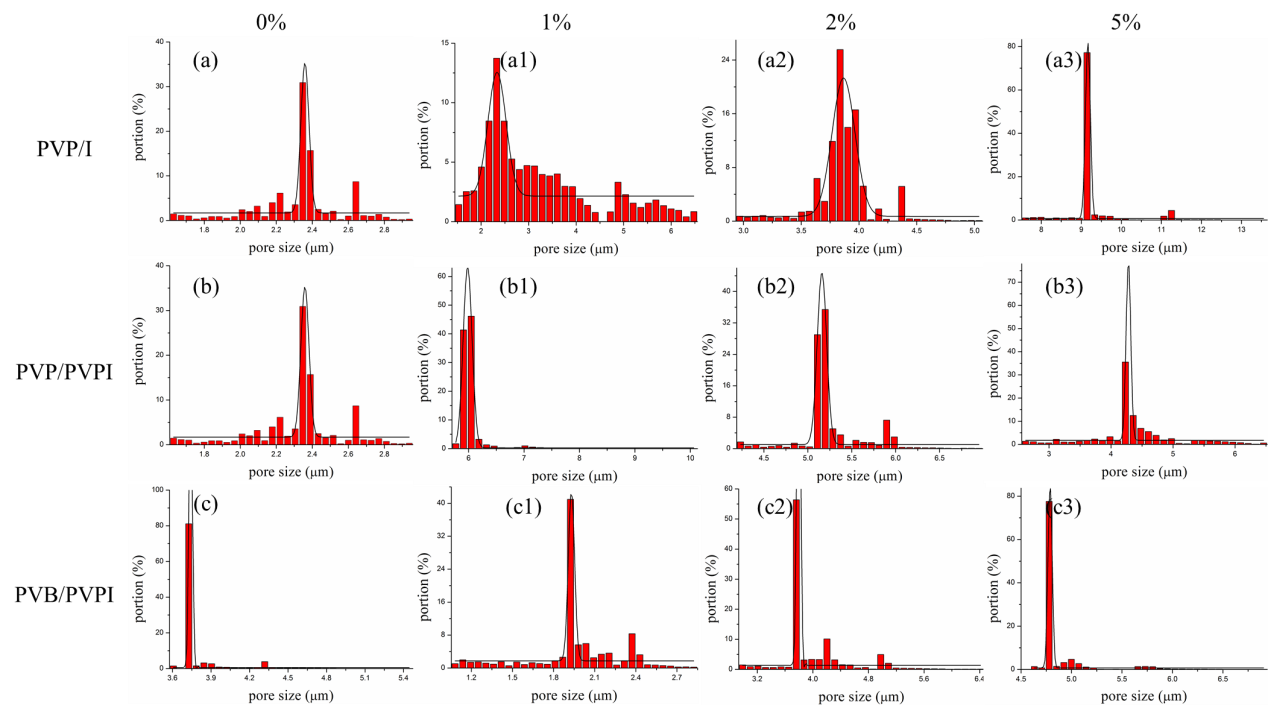


Figure S1 Pore size distribution of the as-spun PVP/I (a-a3), PVP/PVPI (b-b3) and PVB/PVPI(c-c3) fibrous mats with concentration of I/PVPI 0%, 1%, 2% and 5%, respectively.


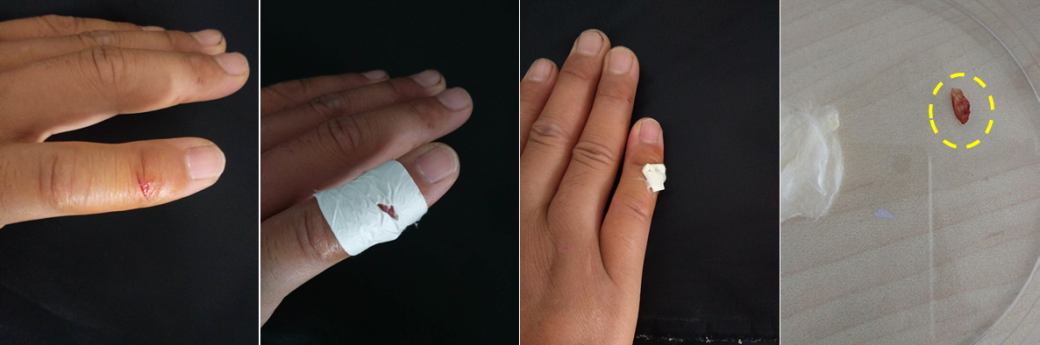


Figure S2 Electrospun PVP-I meshes onto human injured finger, can stem the bleeding quickly, and then heal the wound well.


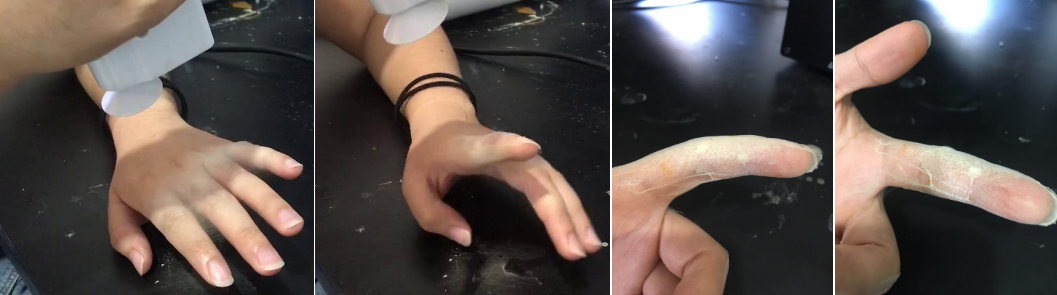


Figure S3 In situ electrospun PVP/I meshes onto human hand and finger, the as-spun meshes showed good conformability on the finger.
